# Supplementary material for: The Histamine H4 Receptor Participates in the Anti-Neuropathic Effect of the Adenosine A3 Receptor Agonist IB-MECA: Role of CD4+ T Cells
Source: Biomolecules. 2021 Oct 2;11(10):1447. doi: 10.3390/biom11101447 (PMC8533073; doi:10.3390/biom11101447)
Supplement: Supplementary file 1 [file biomolecules-11-01447-s001.zip › biomolecules-1335486-supplementary.pdf]

Supplementary Materials

Semi-Quantitative RT-PCR of H4 Receptor

Total RNA was isolated from spinal cord according to the manufacturer's protocol (NucleoSpin RNA II; Machery-Nagel, Bethlehem, PA, USA). Concentration and purity were assessed by spectrophotometric analysis (Genequant-Pro, GE Healthcare, Milan, Italy). Two μg of total RNA was reverse-transcribed to cDNA with M-MLV Reverse Transcriptase (Omniscript; QIAGEN, Milan, Italy) using random primers in a 20 μL reaction incubated at 25 °C for 5 min followed by 60 min at 42 °C and 5 min at 70 °C. Two hundred ng of cDNA was amplified as follows: 30 s of denaturation at 94 °C, 30 s of annealing at 51.2 °C and 30 s of extension at 72 °C for 35 cycles. Primer sequences are the following: mH4R-F 5′-GGT CAT CTT AGC CTT TGT GG-3′; mH4R-R 5′-TTT GTG TTC GTG CTG TTC TT-3′ (Invitrogen, MA, USA). Amplification products (396 bp) were highlighted with ethidium bromide on 1.5% agarose gel. The intensities of the bands were quantified by densitometric analysis.

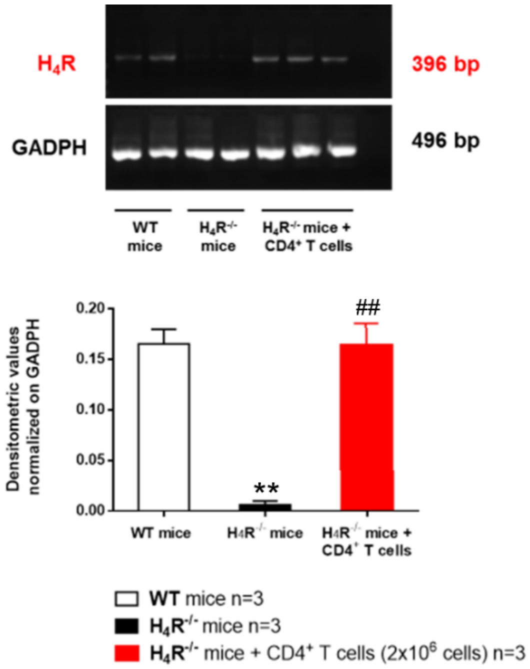

Figure S1. H_4R expression in spinal cord of $H_4R^{-/-}$ mice after $CD4^+$ T cells transfer. Twenty-four hours after $CD4^+$ T cells transfer, mice were sacrificed to analyse H_4R expression in the mouse spinal cord by RT-PCR analysis The same analysis was performed in WT and $H_4R^{-/-}$ mice that did not receive the intravenously injection of $CD4^+$ T cells. ** $p < 0.01$ vs. WT mice and ## $p < 0.01$ vs. $H_4R^{-/-}$ mice.

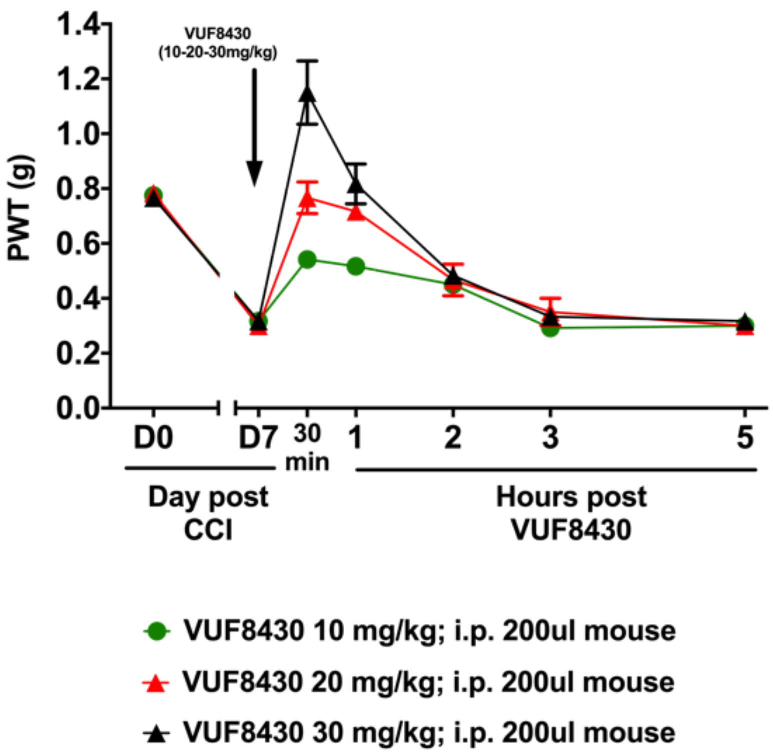

Figure S2. Anti-allodynic effect of H_4R agonist VUF 8430 on CCI-induced neuropathic pain in WT mice. Sciatic nerve ligation was performed 8 days before the acute injection of VUF 8430 (10–30 mg/kg, i.p.). The response to a non-noxious mechanical stimulus was assessed by the Von frey test. Measurements were performed before and 30 min, 1 h, 2 h, 3 h and 5 h after compound administration. Values reported in the graph are referred to the tests conducted on the ipsilateral paw. Data are mean ± SD for 5 mice; * $p < 0.05$ vs. D0 by two-way ANOVA with Dunnett's test; † $p < 0.001$ vs. Day 7 by two-way ANOVA with Tukey's comparisons.
